# MUSIC-BASED INTERVENTION TERMINOLOGY IN ALZHEIMER’S DISEASE AND RELATED DEMENTIAS STUDIES

**DOI:** 10.1093/geroni/igae098.0689

**Published:** 2024-12-31

**Authors:** Sarah McKiddy

**Affiliations:** University of Washington, Seattle, Washington, United States

## Abstract

The surge in interest surrounding music-based interventions (MBIs) for cognitive health, particularly in Alzheimer’s disease and Alzheimer’s disease related dementias (AD/ADRD), has catalyzed interdisciplinary research. However, the collaborative nature of these efforts has led to discrepancies in language usage, hindering the design and interpretation of interventions. In a systematic review conducted by Robb et al. (2018) on the quality of reporting MBI research in healthcare, discordant terminology was identified as an overarching, ongoing barrier to MBI research. The primary aims of this scoping review were to: (1) identify and define the terminology employed in empirical studies describing MBIs within the context of Robb et al.’s (2011) Guidelines for MBIs and studies involving AD/ADRD, (2) elucidate how these terms were operationalized, and (3) propose implications for future research and practice. Included studies showcased a diverse array of MBI strategies in AD/ADRD research, ranging from music therapy practices to social prescribing. However, inconsistencies in terminology usage, such as interchangeably employing terms like “music therapy” and “music intervention,” were observed. This review supports the pressing need for unified terminology that translates despite interdisciplinary boundaries and specialties, facilitating collaboration and advancing both research and practice in MBIs for AD/ADRD. To enhance the clarity of the literature and foster rigor in methodologies, researchers are encouraged to consistently establish and apply clear, operationalized definitions for terminology while also referencing established concepts and seeking consensus within the MBI community.

